# Role of natural products in mitigation of toxic effects of methamphetamine: A review of *in vitro* and *in vivo* studies 

**Published:** 2020

**Authors:** Mohammad Moshiri, Ali Roohbakhsh, Mahdi Talebi, Milad Iranshahy, Leila Etemad

**Affiliations:** 1 *Medical Toxicology Research Center, Mashhad University of Medical Sciences, Mashhad, Iran*; 2 *Department of Clinical Toxicology, Imam Reza Hospital, Mashhad University of Medical Sciences, Mashhad, Iran*; 3 *Pharmaceutical Research Center, Pharmaceutical Technology Institute, Mashhad University of Medical Sciences, Mashhad, Iran*; 4 *Department of Pharmacodynamics and Toxicology, School of Pharmacy, Mashhad University of Medical Sciences, Mashhad, Iran*; 5 *Department of community and Family Medicine, Faculty of Medicine, Mashhad University of Medical Sciences, Mashhad, Iran*; 6 *Department of Pharmacognosy, School of Pharmacy, Mashhad University of Medical Sciences, Mashhad, Iran*

**Keywords:** Addiction, Herbal, Methamphetamine, Toxicity

## Abstract

**Objective:**

Methamphetamine (METH) increases dopamine, norepinephrine and serotonin concentrations in the synaptic cleft, and induces hyperactivity. The current management of acute METH poisoning relies on supportive care and no specific antidote is available for treatment. The main objective of this review was to present the evidence for effectiveness of the herbal medicine in alleviating the adverse effects of METH abuse.

**Materials and Methods:**

Literature search was performed using the following electronic databases: MEDLINE, Scopus, PubMed and EMBASE.

**Results:**

Plant-derived natural products ginseng and sauchinone reduced METH-induced hyperactivity, conditioned place preference and neurological disorder. *Garcinia kola* decreased METH-induced hepatotoxicity, raised METH lethal dose, and restored the METH-impaired cognitive function. Repeated administration of baicalein resulted in attenuation of acute binge METH-induced amnesia via dopamine receptors. Activation of extracellular-regulated kinase in the hypothalamus by levo-tetrahydropalmatine facilitated the extinction of METH-induced conditioned place preference and reduced the hyperactivity. Other herbal medicine from various parts of the world were also discussed including hispidulin, silymarin, limonene, resveratrol, chlorogenic acid and barakol.

**Conclusion:**

Based on the current study, some natural products such as ginseng and levo-tetrahydropalmatine are promising candidates to treat METH abuse and poisoning. However, clinical trials are needed to confirm these finding.

## Introduction

Methamphetamine (METH), with common street names "ice", "jib", "crystal" and "speed", is a potent neurotoxic stimulant and has psychedelic properties . METH has a chemical formula of C_10_H_15_N derived from amphetamine and is available as powder or crystals. Nagai Nagayoshi firstly synthesized this compound from ephedrine in 1983 (Albertson et al., 1999 ▶; Lee, 2011 ▶). According to statistics, the number of METH consumers is increasing every year. The number of METH consumers has been estimated to be approximately 37 million people, in 2015 (United Nations Office on Drugs and Crime, 2017 ▶). METH is generally used by oral ingestion, intravenous injection, nasal insufflations (snorting) or smoking. Depending on the routes of consumption, its bioavailability varies between 60 and 100% (Harris et al., 2003 ▶). METH metabolism in the liver produces less active metabolites, such as amphetamine, 4-hydroxymethamphetamin and norephedrine. Approximately 50% of METH is excreted unchanged in the urine (Cruickshank and Dyer, 2009 ▶).

METH indirectly affects serotoninergic, dopaminergic and adrenergic systems. Due to similar molecular structure, METH inhibits dopamine, norepinephrine and serotonin transporters reuptake and increases their concentrations in the synapses and indirectly stimulates monoamine receptors (Courtney and Ray, 2014 ▶; Moshiri et al., 2018 ▶). METH-induced hyperactivity (MIH) is resulted from release and inhibition of the reuptake of dopamine (Kim et al., 1998 ▶). Repeated administration of METH causes an enhancement in the METH accelerated motor effects. This phenomenon is named sensitization or reverse tolerance (RT) (Kim et al., 1996 ▶; Tsang et al., 1985 ▶). It was reported that treatment of animals with METH increases sensitivity to apomorphine, a dopamine receptor agonist, and induces dopamine receptor super sensitization (DRS) (Tsang et al., 1985 ▶). The enhanced neuronal dopaminergic transmission in mesolimbic tissue of the brain has a very important role in behavioral changes due to different stimulants including METH (Bello et al., 2011 ▶).

Conditioned place preference (CPP) paradigm is an established method for evaluating the potential reinforcing properties of drugs, and is a model for assessing psychological dependence (Alavi et al., 2016 ▶; Bahi et al., 2008 ▶; Duncan et al., 1983 ▶; Finlay et al., 1988 ▶; Kim et al., 1998 ▶; Wang et al., 2010b ▶). CPP and behavioral sensitization of all drugs, specially stimulants, are referred to elevated dopamine concentration and DRS (Kim et al., 1998 ▶). Following chronic consumption of METH or other amphetamine derivatives, depletion of dopamine in action sites results in CPP (Duncan et al., 1983 ▶). 

Low to moderate doses of METH may cause nausea, vomiting, headache, tremor, irritability, euphoria, positive mood, increased temperature, reduced appetite, confusion, hallucinations, behavioral disinhibition and short-term improvement of cognition and formication (Moshiri et al., 2019 ▶; Vahabzadeh and Ghassemi Toussi, 2016 ▶). Sever toxicity may be associated with renal failure, seizure and coma. Serotonin syndrome may occur in a small proportion of users (Kiyatkin and Sharma, 2009 ▶). Chronic exposure to METH can lead to paranoid psychosis, hallucinations, and cardiomyopathy and increases the risk of hemorrhagic and ischemic stroke (Cruickshank and Dyer, 2009 ▶).

The current management of acute METH poisoning relies on supportive care and no specific antidote is available for treatment. Benzodiazepines are used to control delirium, agitation, and seizures. In patients who do not respond to benzodiazepines, haloperidol or other antipsychotic drugs may be considered (Tien et al., 2010 ▶). It was suggested that compounds which decrease the availability of monoamines in presynaptic regions, block the monoamine receptors or suppress the monoamines post receptor signaling, are not able to attenuate the METH-induced reinforcement and adverse effects (Ghadiri et al., 2017 ▶; Pickens et al., 1968 ▶).

The main mechanisms proposed for METH-induced neurotoxicity are oxidative stress, monoamines and ionic homeostasis dysregulation and hyperthermia. Oxidative stress is mediated by two distinct oxygen and nitric oxide (NO)-based pathways. So, the antioxidant compounds and NO synthase (NOS) inhibitors have shown protective effects on METH-induced neurotoxicity (Jang et al., 2012b ▶). 

Herbs have been used in traditional medicine to cure a broad spectrum of illnesses over the past 1000 years. Information about medicinal plants and their therapeutic applications have been preserved in Iranian, Indian and Chinese traditional medicine compendium(Taleb et al., 2014 ▶). 

Plants produce a wide range of metabolites in their roots, leaves, flowers, or seeds that are frequently used in the pharmaceutical field. Plant-based medicines are more affordable and available than modern medicines and have various pharmacological properties including anti-inflammatory, antimicrobial, antifibrotic, anticancer, and neuroprotective effects. These health benefits are attributed to several classes of phytochemicals such as flavonoids, polyphenols, tannins, alkaloids, and monoterpenes (Umesha et al., 2013 ▶).

Herbal medicines have been used to reduce the adverse effects, dependency and craving and manage withdrawal syndrome of drugs since ancient times (Lu et al., 2009 ▶). The effectiveness of herbal medicines in the treatment of addiction to nicotine, alcohol, opiate, cocaine and METH was evaluated in *in-vitro*, *in-vivo* and clinical studies (Mantsch et al., 2007 ▶; Wang et al., 2010b ▶; Wen et al., 2014 ▶; Yang et al., 2008 ▶) 

 The aim of this review article is to explore and identify the role of herbal-based medicines in the treatment of METH abuse and toxicity.

## Materials and Methods

We performed a non-systematic literature review from several databases including Scopus, PubMed and EMBASE. We searched the literature without time restriction. Searches were conducted using the keywords “Addiction”, “Herb”, “Methamphetamine”, “Toxicity”, poison”, “natural” and “extract”. More than 300 articles were found and 42 related articles were added. No human studies such as case reports or clinical trials, were found.

## Results

**Ginseng**

Ginseng is a perennial plant with fleshy roots that belongs to the Araliaceae family. This popular herb grows widely in America and more tropical areas, especially, east of Asia and oriental countries. Over many years, in traditional medicine, ginseng roots have been used as anti-diabetes, anti-inflammatory, antianxiety, anti-fatigue, anti-depressant, and memory enhancer, and for improvement of physical and sexual activities (Lacaille-Dubois and Wagner, 1996 ▶).

Ginseng prevented the development of morphine tolerance and dependence in rodents (Kim et al., 2005 ▶; Tokuyama and Takahashi, 2001 ▶). It also reduced morphine-, cocaine-, and METH-induced RT (Tokuyama and Takahashi, 2001 ▶). In addition, ginseng reduced METH- and cocaine-induced hyperstimulation even after 30 days of discontinuation (Tokuyama et al., 1996 ▶). 

In recent years, scientists have investigated the ability of secondary metabolites from ginseng to ameliorate the METH adverse effect (Table 1). 

**Table 1:** Summarized effects of ginseng on methamphetamine adverse effects. All experiences were performed in mice

**Author [ref.]**	**Evaluation **	**Design**	**Results**
Kim (Kim et al.)	RT	GTS (100 or 200 mg/kg, PrT) + METH (2 mg/kg) other day	Reduced by 200 mg/kg GTS but not by 100 mg/Kg GTS
	DRS hypothermic response to AP	24 hours after RT received AP (1 mg/ kg) repeated every 30 min (4 mg/kg)	Inhibited by 200 mg/kg GTS, but not by 100 mg/kg GTS
	Enhanced ambulatory activity of AP		Reduced by 200 mg/kg GTS but not by 100 mg/Kg GTS
Kim (Kim et al., 1996 ▶)	MIH	GTS (100 or 200 mg/kg, PrT) + METH (2 mg/kg)	Reduced by 200 mg/kg GTS but not by 100 mg/Kg GTS
	CPP	GTS (50 or 100 mg/kg, IP, PrT) + METH (2 mg/kg)	Reduced by 100 mg/kg GTS but not by 50 mg/Kg GTS
	DRS	24 hours after CPP received AP (2 mg/kg, SC)	Reduced by 100 mg/kg GTS but not by 50 mg/Kg GTS
	AP induced climbing behavior	GTS (50, 100, 200 mg/kg IP, PrT) + AP (2 mg/kg)	Reduced by 200 and 100 mg/kg GTS but not by 50 mg/Kg GTS
Oh (Oh et al., 1997 ▶)	Strial DA, DOPAC, HVA	GTS (50 and 100 mg/kg, IP, PrT, 2 times) + METH (10 mg/kg, 4 times)	Restored catecholamines depletion, 100 mg/kg was more potent than 50 mg/kg
Kim (Kim et al., 1998 ▶)	MIH	Rb1 or Rg1 (50, 100 and 200 mg/kg, IP, PrT) + METH (2 mg/kg IP)	Reduced by 100 and 200 mg/kg; not by 50 mg/kg
	CPP	Rb1 or Rg1 (50, 100 and 200 mg/kg, IP, PrT) + METH (2 mg/kg IP)	Reduced by 100 mg/kg; not by lower doses
	DRS	24 hours after CPP received AP (2 mg/ kg)	Reduced by 100 mg/kg ; not by lower doses

AP=apomorphine, CPP=conditional place performance, DA=dopamine, DOPAC=3, 4-dihydroxyphenylacetic acid, GTS=ginseng total saponin, HVA=homovanillinic acid, METH=methamphetamine, MIH=methamphetamine-induced hyperactivity, PrT=pretreatment, and RT=reverence tolerance.

Pseudoginsenoside-F11 (PF11) is an ocotillol-type saponin found in *Panax quinquefolius *(American ginseng). The researchers showed that PF11 prevented and treated the METH-induced neurological disorders (Fu et al., 2016 ▶; Wu et al., 2003 ▶). The orally administrated PF11 greatly reduced the anxiety-like behaviors of mice and rats in the light–dark box task. Oral administration of PF11 also shortened the METH-induced prolonged immobility time in the forced swimming task and increased latency in Morris water maze task which implied that it decreased the depression-like behavior and memory dysfunction (Wu et al., 2003 ▶). By measuring the neurotransmitters concentrations, it was observed that PF11 antagonized METH-induced decrease of dopamine and other neurotransmitters (Wu et al., 2003 ▶). Although, PF11 is not produced by *Panax ginseng*, it was documented that repeated pretreatment with *P. ginseng* extract inhibited development of RT and reappearance of behavioral sensitization to METH and cocaine, which are known as typical effects of psychostimulants (Kim et al., 2005 ▶). 

**Figure 1:**
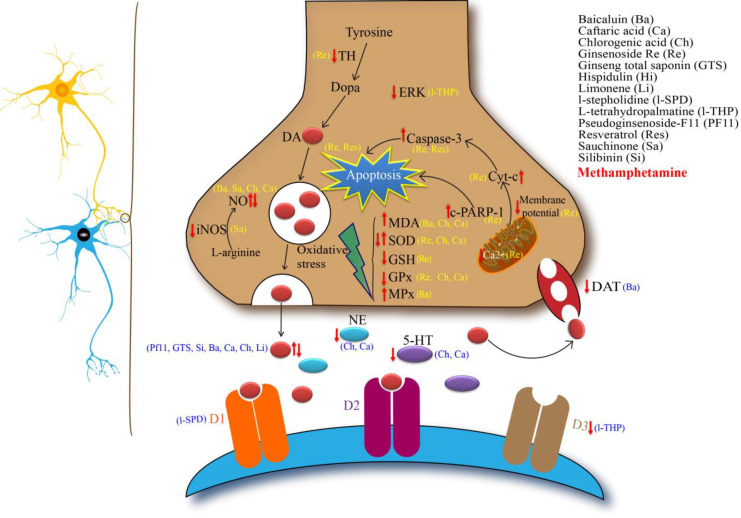
A schematic illustration of therapeutic mechanisms of different natural compounds in the treatment of methamphetamine-induced neurotoxicity.  , decrease and  , increase following methamphetamine treatment.    means that both effects were reported in different studies. Yellow and blue abbreviations denote the related compounds in the right section of the Figure (ref)

Active compounds of ginseng have also shown similar effects. For example, it was demonstrated that ginseng total saponin (GTS) inhibited METH-induced hyperlocomotion, CPP and RT, in rats and mice (Kim et al., 2005 ▶; Kim et al., 1996 ▶; Tokuyama and Takahashi, 2001 ▶). However, administration of GTS alone had no effect on CPP (Tokuyama et al., 1996 ▶). As mentioned in the introduction, METH-induced CPP is resulted by DRS. GTS inhibited development of DRS and exhibited antidopaminergic properties (Kim et al., 1996 ▶). It was suggested that GTS modulated the dopaminergic system activity by reducing the reuptake of dopamine and raising its content in the rat brain (Figure 1) (Lacaille-Dubois and Wagner, 1996 ▶). GTS also modulated the action of serotonergic/adenosine A2A/δ-opioid receptor complex (Kim et al., 2005 ▶).

Chronic use of METH was reported to induce depletion of striatal dopamine and its metabolites. Pretreatment of METH- intoxicated mice with GTS (100 mk/kg) for one-week, resulted in a marked reduction in striatal dopamine, 3, 4-dihydroxyphenylacetic acid, and homovanillinic acid. However, treatment of mice with GTS alone did not change the level of striatal dopamine and its metabolites. So, the researchers suggested that GTS might prevent METH-induced Parkinsonism (Oh et al., 1997 ▶). 

Ginsenosides and panaxosides play an important role in therapeutic effects of ginseng. Ginsenosides attenuated the morphine-induced cAMP signaling pathway (Kim et al., 2005 ▶). Ginsenoside Re is the main ginsenoside that is found in ginseng leaf, berry and root. The results of *in vitro* and *in vivo* studies showed that ginsenoside Re significantly alleviated METH-induced neurotoxic through enhancement of the antioxidant capacity, prevention of mitochondrial oxidative damage, mitochondrial translocation of protein kinase C, and apoptosis in the dopaminergic system in mouse brain and SH-SY5Y neuroblastoma cells (Figure 1) (Nam et al., 2014 ▶; Shin et al., 2014 ▶). Ginsenosides Rb1 and Rg1 are the major components of GTS. Both single and repeated administration (100 and 200 mg/kg) of these ginsenosides inhibited the hyperlocomotion and CPP following METH administration. These components were also able to attenuate DRS. However, lower doses of both compounds (50 mg/kg) were not effective against CPP, MIH, or DRS in mice (Kim et al., 1998 ▶). 

It was proposed that ginseng extract can be a useful alternative in prevention of METH and cocaine addiction (Kim et al., 2005 ▶; Tokuyama et al., 1996 ▶). Ginseng can also effectively attenuate psychostimulants-induced tolerance and dependence and prevent their adverse effects. However, clinical trials are essential to confirm these results (Table2).

***Garcinia kola***

*Garcinia kola*, from Clusiaceae/ Guttiferae family, is a well-known herb due to its wide range of therapeutic uses. In liver injury induced by METH, oral administration of the aqueous extract of *G. kola* for 6 weeks, attenuated the raised serum levels of liver marker enzymes, alkaline phosphatase, aspartate aminotransferase, and alanine aminotransferase, as well as total bilirubin. The pathological findings with a significant reduction in hepatic vacuolated cells, confirmed the biochemical laboratory test results. None of METH-intoxicated animals pretreated by 200 mg/kg of aqueous extract of *G. kola* (AEGK) died. Although, AEGK at doses of 100 and 0 (control group) mg/kg respectively caused 30% and 50% death in METH-intoxicated animals. Thus, it was suggested that *G. kola* increased median lethal dose of METH (Oze et al., 2010 ▶). Therefore, *G. kola* was proposed as a hepatoprotective plant for the treatment of METH-induced liver toxicity (Oze et al., 2010 ▶). 

It was also reported that kolaviron, a biflavonoid complex from *G. kola* seeds, can delay or even attenuate the onset of stereotypic behaviors in mice treated with a single dose of METH and inhibited the negative effects of METH on learning and memory (Ijomone et al., 2012 ▶). The brain histological findings revealed that pre-treatment with kolaviron protected the hippocampal CA1 and CA2 regions against the METH-induced neurotoxicity (Ijomone et al., 2012 ▶). The researchers suggested that kolaviron restored the impaired cognitive function induced by METH through inhibition of acetylcholinesterase (Figure 1) (Table 2) (Ijomone and Obi, 2013 ▶).

***Clerodendrum inerme*** **L.**

*Clerodendrum inerme* L. (Family: Verbenaceae) has been traditionally used for the treatment of scrofulous, chronic pyrexia, asthma, bronchitis, inflammation and epilepsy (Shukla et al., 2014 ▶). In an animal study, the effects of ethanolic extract of *C. inerme* leaves on METH–induced changes in locomotion, motor coordination, muscle power, and prepulse inhibition of acoustic startle response (PPI) were evaluated in mice. The ethanolic extract significantly inhibited hyperlocomotion and prevented the PPI disruptions induced by METH, however ,the extract did not affect motor coordination and muscle tone (Chen et al., 2012a ▶). Later, it was shown that the active constituent from the *C. inerme* ethanolic extract namely hispidulin also decreased METH-induced hyperlocomotion, nevertheless hispidulin neither impairs motor tone nor induces sedation in mice (Huang et al., 2015 ▶; Liao et al., 2016 ▶). Intracerebellar microinjection of hispidulin also inhibited the METH-induced hyperlocomotion through activation of alpha6 subunit-containing GABAA receptors (Figure 1) (Table 2) (Liao et al., 2016 ▶).

***Silybum marianum***

*Silybum marianum* (milk thistle) belongs to the Asteraceae family has been traditionally used in treatment of liver diseases (Gholami et al., 2015 ▶; Karimi et al., 2011 ▶). Silibinin (silybin) is the most prevalent and active component isomer in silymarin. Silymarin flavonoid complex is derived from the seeds and fruits and has many medical benefits (Gholami et al., 2016 ▶; Gholami et al., 2015 ▶; Karimi et al., 2011 ▶). In recent advances, silibinin showed a preventive effect on METH-induced recognition memory impairment in novel object recognition test (NORT) in animal (Lu et al., 2010 ▶). Silibinin significantly increased the object recognition index in METH-injected mice. The neuroprotective effects of silibinin were associated with improvement of serotonin and dopamine depletion in the hippocampus and the prefrontal cortex, respectively (Figure 1) (Table 2) (Lu et al., 2010 ▶).

**Table 2:** Summarized effects of herbs on methamphetamine (METH) intoxication and adverse effects

**Effective against METH adverse effect**	**Compound**	**Herb**
MIH	Barakol	*C. siamea* Lamk
MIH Prepulse inhibition of acoustic startle response	Ethanolic extract	*Clerodendrum inerme* L.
MIH	hispidulin	
METH increased oxidative stress indexes METH–induced hepatotoxicity	Chlorogenic acid	Coffee
MICPP (all parameters) MIH	L-tetrahydropalmatine	*Corydalis ternata*
METH induced hepatotoxicity METH lethal dose	Aqueous extract	*Garcinia kola*
METH-impaired cognitive function METH-impaired learning and memory METH-induced hippocampal neurotoxicity METH stereotypic movement	Kolaviron biflavonoid	
METH-increased dopamine concentration in NA MIH	Limonene	*Citrus*
Freezing time in the forced swimming task Latency in Morris water maze METH induced anxiety-like behavior	Pseudoginsenoside-F11	Ginseng
DRS METH induced parkinsonism MICPP MIH RT	ginseng total saponin	
DRS METH-induced neurotoxicity MICPP MIH	Ginsenosides	
METH-increased dopamine concentration in NA MIH	Radix methanol extracts	*Glycyrrhizae uralensis*
METH induced [3H] dopamine overflow MIH	Resveratrol	Grapes
METH induced dopaminergic neurotoxicity METH-reduced Nitric Oxide production METH-increased gelial cells activation MICPP MIH	Sauchinone	*Saururus chinensis*
Harm reduction of neutrophil in striatal METH-reduced dopamine transporter in striatal METH-reduced dopamine level in striatal METH-induced memory deficits METH-induced lipid peroxidation METH-reduced Nitric Oxide production	Baicalein	*Scutellariae baicalensis *Georgi
METH-impaired memory recognition METH-induced hippocampal and prefrontal cortex	silibinin	*Silybum marianum*
METH self-administration behavior MIH	l-stepholidine	*Stephania intermedia*
METH induced anxiety like behavior MICPP	l-Scoulerine	

DRS=dopamine receptor super sensitization; METH=Methamphetamine; MICPP=METH-induced conditioned place preference; MIH=METH-induced hyperactivity; NA=nucleus accumbens; RT=sensitization or reverse tolerance.

***Scutellariae baicalensis*** **Georgi**

*Scutellariae baicalensis* Georgi (or Huang Qin) is a Chinese plant that belongs to the Lamiaceae family and has a variety of therapeutic applications. Baicalein, an active component isolated from the roots possesses free radical scavenging and anti-inflammatory effects. Several *in vitro* and *in vivo* studies reported potent neuroprotective properties for baicalein (Sowndhararajan et al., 2017 ▶). A recent research showed that baicalein improved METH-induced memory deficits in mice. Repeated administration of baicalein resulted in attenuation of acute binge METH-induced amnesia via dopamine D2 receptors in the passive avoidance test (Figure 1). It also decreased lipid peroxidation and peroxynitrite production in the hippocampus of mice (Wong et al., 2014 ▶). Pretreatment of METH-intoxicated mice with baicalein, attenuated the loss of dopamine transporter (Wu et al., 2006 ▶) and dopamine level (Liu et al., 2006 ▶) in the striatum, dose-dependently. The authors suggested that the neuroprotective effect of baicalein in the mouse striatum is related to reduction in neutrophil count and lipid peroxidation (Sowndhararajan et al., 2017 ▶; Wu et al., 2006 ▶). Baicalein also elevated NO level in the brain and protected mice brain from METH-induced reduction of NO content (Liu et al., 2006 ▶). 

**Sauchinone**

Sauchinone is an active compound of *Saururus chinensis* (Asian lizard's tail) that has been used for the treatment of hepatitis and jaundice in Chinese traditional medicine (Kim et al., 2013a ▶). Sauchinone, which is a lignan structure, has hepatoprotective, antioxidant and anti-inflammatory effects. It can suppress NO production in dorsal striatum of mouse (Jang et al., 2012b ▶; Lee et al., 2003 ▶). Regarding the effect of NO on METH-induced neurotoxicity, the potential protective effect of sauchinone against METH adverse effects, was evaluated (Jang et al., 2012a ▶). Sauchinone attenuated the METH-induced degeneration of dopaminergic nerve terminals. In addition, pretreatment with sauchinone reduced the glial cell activation markers (glial fibrillary acidic protein and CD11b antigens) and inhibited the synthesis of NO (Figure 1) (Jang et al., 2012b ▶). However, administration of sauchinone alone did not show any significant changes in glial markers or NO synthase activity (Jang et al., 2012b ▶). 

Kim and coworkers evaluated the effects of pretreatment with sauchinone on METH-induced hypertactivity (MIH) and CPP. Sauchinone induced a dose-dependent protective effect on MIH and suppressed the expression and acquisition of METH-induced CPP (MICPP) (Kim et al., 2013a ▶). The authors believed that these effects of sauchinone were related to inhibition of NO synthase (Table 2). 

**Levo-tetrahydropalmatine**

Since last centuries, many species of *Corydalis* have been used as traditional herbal medicines in Asia. Levo-tetrahydropalmatine (L-THP) is one of the most important active ingredients of *Corydalis ternata* and it has analgesic (Wang et al., 2010a ▶), sedative, hypnotic, and neuroleptic properties (Ding, 1987 ▶; Zhao et al., 2014a ▶). It was approved by ministry of health of China for medical uses and has been implemented into practice since 1977 (Gong et al., 2016 ▶; Su et al., 2013 ▶). L-THP is an antagonist of D1 and D2 dopamine receptors and modulates D3 receptors (Wang and Mantsch, 2012 ▶; Zhao et al., 2014a ▶). Its affinity for D1 is 4-7 fold higher than for D2 receptor (Guo et al., 1997 ▶). The rewarding properties of addictive drugs have been attributed to both D1 and D2 receptors (Bardo et al., 1993 ▶; Park et al., 2014 ▶; Shippenberg and Herz, 1987) (Figure 1). So, the therapeutic effects of L-THP against different kinds of drug addiction such as nicotine (Faison et al., 2016 ▶), opiate (Liu et al., 2005 ▶; Liu et al., 2009 ▶; Yue et al., 2012 ▶), cocaine (Mantsch et al., 2007 ▶; Mantsch et al., 2010 ▶; Sushchyk et al., 2016 ▶; Wang and Mantsch, 2012 ▶; Xi et al., 2007 ▶) and ethanol (Kim et al., 2013b ▶) have been evaluated. Additionally, L-THP significantly ameliorated heroin craving and increased the abstinence rate of heroin abusers in a double-blinded and placebo-controlled clinical trial (Yang et al., 2008 ▶). L-THP pretreatment attenuated the acquisition and expression of MICPP, facilitated the extinction of MICPP and prevented the reinstatement of MICPP in mice and rats (Gong et al., 2016 ▶; Su et al., 2013 ▶) (Table 3). However, administration of L-THP alone could not induce any conditioned place preference (Gong et al., 2016 ▶; Su et al., 2013 ▶). Yun et al. (2014) ▶ also reported that L-THP suppressed the serotonin-induced head twitch response (HTR) and climbing behavior through the activation of dopaminergic system in mice (Yun, 2014a ▶).

**Table 3:** Summarized of evaluation the effects of l-tetrahydropalmatine (L-THP) on methamphetamine (METH) adverse effects

**Reference**	**Animal**	**Evaluation**	**Design**	**Results**
Yun (Yun, 2014a ▶)	Mice	Time spent in climbing behavior	PrT l-THP 10 and 15 mg/kg, i.p.) + APO (2 mg/kg)	Inhibited climbing behavior
		HTR	PrT l-THP 10 and 15 mg/kg, i.p.) + 5-HT (80 g/10 l/mouse, i.c.v.)	Inhibited HRT
	Rats	MIH	l-THP (10 and 15 mg/kg) + METH (1 mg/kg ip)	Inhibited MIH
		D3 receptor mRNA expression in CPU (PCR)	l-THP (15 mg/kg) + METH (1 mg/kg ip)	Block the METH-induced decrease in D3 receptor mRNA
Gong (Gong et al., 2016 ▶)	Rats	METH Self-administration	METH addicted rats PrT l-THP (0, 1.25, 2.5 and 5 mg/kg, i.p.)	Decreased the number of active nose pokes
		Reinstatement test	L-THP (0, 1.25, 2.5 and 5 mg/kg, i.p.) + METH (1 mg/kg) I.P.	Decreased the number of active nose pokes
		MIH	PrT l-THP (0, 1.25, 2.5 and 5 mg/kg, i.p.) + METH (1 mg/kg, i.p.).	5 mg/kg reduced total distance travel
Su (Su et al., 2013 ▶)	mice	MICCP A) acquisition	PrT L-THP (5, 10 and 20 mg/kg) + METH (1 mg/kg, i.p.) in conditioning phase	Attenuated by 10 and 20 mg/kg
		B) expression	PrT L-THP 5, 10 and 20 mg/kg) + METH in behavioral test	Attenuated by 5 and 10 mg/kg
		C) extinction	PrT METH (1 mg/kg i.p.) + L-THP (10 mg/kg) in the testing phase	Facilitate the extinction of MICPP
		Reinstatement	PrT L-THP (10.0 mg/kg.) + METH (1 mg/kg, i.p.).	inhibit the reinstatement
Chen (Chen et al., 2012b ▶)	mice	Learning and memory (Morris water maze test) Escape latencies Platform site crossings	METH (5 or 10 mg/kg) + L-THP (5 or 10 mg/kg)	Resolved METH -elongation escape latencies and METH-reduced platform site crossings
	Expression of total ERK1/2 in the PFC (Western blotting)	METH (10 mg/kg) + L-THP (10 mg /kg )	Reversing METH- reduction ERK 1/2 expression
Zhao (Zhao et al., 2014a ▶)	mice	MIH	METH (2 mg /kg) + l-THP (5 and 10 mg/kg)	l-THP attenuated MIH
		Development and Expression of MILS	METH (2 mg /kg) + l-THP (5 and 10 mg/kg)	l-THP attenuated the Development and expression of MILS
		Activation of ERK (ERK1/2 in the NAc and CPU)	PrT l-THP (5 and 10 mg/kg) + METH (2 mg/kg)	l-THP attenuated METH-induced Phosphorylation of ERK1/2

APO=apomorphine; CPU=caudate putamen; D=dopamine; ERK=extracellular-regulated kinase; HTR=5-HT-induced head twitch response; i.c.v.=Intracerebroventricular injection; i.p.=Intraperitoneal; l-THP=l-tetrahydropalmatine; METH=methamphetamine; MICPP=METH-induced conditioned place preference; MIH=methamphetamine induced hyperactivity; MILS=Meth-induced locomotor sensitization; NAC=nucleus accumbens; PFC=prefrontal cortex; PrT=Pretreatment with; Sch-23390=dopamine 1 antagonist .

L-HTP did not affect locomotor activity of animals, therefore, the HTR suppressing effect of L-THP was not related to motor impairments (Yue et al., 2012 ▶).

In addition, L-THP attenuated the METH-induced hyperlocomotion and expression of the D3 receptor mRNA in the striatum (Yun, 2014a ▶). Zhao and coworkers also reported that co-administration of L-THP and METH diminished METH-induced locomotor sensitization and hyperactivity (Zhao et al., 2014a ▶). 

Extracellular-regulated kinase (ERK) is an important protein that contributes to synaptic plasticity and memory formation (Figure 1) (Thiels and Klann, 2001 ▶). The reduction in ERK activation following METH administration was associated with recognition memory impairment (Kamei et al., 2006 ▶). Zhao et al. showed that L-THP could activate the ERK signaling pathway in the nucleus accumbens (NAc) and caudate putamen (CPu) (Zhao et al., 2014a ▶). Addictive drugs increase the phosphorylation of ERK (Valjent et al., 2006a ▶). Amphetamine-induced locomotor sensitization and MICPP were attenuated by inhibition of ERK phosphorylation (Valjent et al., 2006a ▶; Valjent et al., 2006b ▶). It was also reported that single injection of L-THP to alcohol naïve mice increased phosphorylation of ERK in the CPu but not in the NAc (Kim et al., 2013b ▶). 

In addition to inhibitory effects of L-THP on dopamine receptors and ERK activation, L-HTP inhibited the amygdaloidal dopamine release (Lin et al., 2002 ▶), facilitated the attachment of GABA to GABAA receptors (Halbsguth et al., 2003 ▶), antagonized the α1 adrenergic receptors (Ko et al., 1996 ▶), modulated serotonin receptors (Liu et al., 2009 ▶), inhibited voltage-sensitive Ca^2+^ channels (Henkes et al., 2011 ▶) and inhibited the expression of K⁺ channel (Kv1.5) (Li et al., 2017 ▶). All of these functions are involved in the development of addiction and dependence to drugs (Volkow and Morales, 2015 ▶). Therefore, it seems that L-THP is an appropriate compound for attenuation of METH adverse effects. It also has potential to be used in the treatment of METH abuse. However, more clinical trials are warranted to support its applications in clinical practice. 

***Stephania intermedia***

l-stepholidine (l-SPD) is an alkaloid isolated from *Stephania intermedia* (Family: Menispermaceae). l-SPD possesses dual pharmacological effects on dopamine receptors. It acts as a partial agonist at D1 receptors and an antagonist at D2 receptors (Natesan et al., 2008 ▶). It was reported that l-SPD effectively antagonized the D1 receptors in the presence of METH-induced dopamine release and attenuated METH self-administration behavior in rats (Yue et al., 2014 ▶). Yue and coworkers assessed the effect of l-SPD on METH-induced locomotor sensitization in mice. They found that l-SPD prevented the hyperlocomotion and sensitized motor behaviors induced by acute and chronic METH administration (Ma et al., 2014 ▶). Their findings suggested that l-SPD may be utilized therapeutically for the treatment of METH dependence. However, the poor bioavailability and high production costs of 1-SPD are noted as the limitations in clinical researches (Sun et al., 2009 ▶). Mi et al. evaluated the anti-addictive properties of l-scoulerine (l-SLR), an analogue of l-SPD. l-SLR is a D2 receptor antagonist, D1 receptor agonist and a 5-HT1A receptor partial agonist (Figure1) (Mi et al., 2016 ▶). l-SLR attenuated the METH-induced anxiety-like behaviors in zebrafish. Indeed, pretreatment of mice with l-SLR attenuated chronic METH-induced behavioral sensitization and blocked the expression of METH-induced CPP. However, L-SLR failed to reduce acute MIH (Table 2) (Mi et al., 2016 ▶). 

**Other herbal compounds**

There are more compounds from several herbs that have been evaluated on METH-induced adverse effects in independent researches or as a part of an experimental method. Accordingly, barakol, the major constituent of *Cassia siamea* Lamk, dose-dependently decreased the METH-induced hyperlocomotion via inhibition of dopaminergic receptors (Sukma et al., 2002 ▶).

Chlorogenic acid is another important polyphenol found in coffee, black tea, eggplants, peaches, prunes, green tea, and several other foods (Rio et al., 2010 ▶). Chlorogenic acid has anti-inflammatory, antioxidant and hepatoprotective effects. Pretreatments of rats with chlorogenic and caftaric acids resolved the METH–induced hepatotoxicity and reversed METH-increased oxidative stress indexes (Koriem and Soliman, 2014 ▶).

Resveratrol (RES) is a polyphenolic compound of grapes with antioxidant and anti-inflammatory properties that attenuated dopamine depletion in an animal model of Parkinson's disease induced by 6-hydroxydopamine (Khan et al., 2010 ▶). Although, pretreatment of mice with single administration of RES did not reduce acute MIH, repeated administration of RES (1–20 mg/kg) decreased MIH. Multiple-dose administration of RES also attenuated METH-induced dopamine overflow in the rat brain (Figure 1) (Miller et al., 2013 ▶). It also protected neuronal cell lines against METH-induced apoptotic by caspase-3 dependent pathway (Kanthasamy et al., 2011 ▶).

Limonene a monoterpene from genus *Citrus* (Zhou et al., 2009 ▶) showed anxiolytic-like and antinociceptive properties and regulatory effects on different neurotransmitters (De Almeida et al., 2012 ▶; Do Amaral et al., 2007 ▶; Zhou et al., 2009 ▶). The HTR response was dose-dependently inhibited by intracerebroventricular injection of limonene in mice (Yun, 2014b ▶). Pretreatment of rats with limonene (200 and 400 mg/kg), also reduced MIH in a dose-dependent manner (Yun, 2014b ▶). The protective effect of limonene on MIH may be related to increases in dopamine levels in the nucleus accumbens (Yun, 2014b ▶) and GABA levels in rat brain (Figure 1) (Zhou et al., 2009 ▶). 

The bioactive components from *Glycyrrhizae uralensis* radix with neuroprotective properties were able to suppress cocaine-induced dopamine release. Oral pretreatment of METH-intoxicated rats with methanolic extract of *G. uralensis* radix dose-dependently reversed MIH. Moreover, the methanolic extract completely reversed the increased dopamine content in the brain tissues following METH treatment (Zhao et al., 2014b ▶). 

Curcumin is a major natural phenolic component of *Curcuma longa* that has shown antidepressant effect. Neurochemical studies revealed that curcumin has the ability to modulate the serotonin pathway dependent function (Kulkarni et al., 2008 ▶). Curcumin also dose-dependently attenuated opioid tolerance dependence through suppressing Ca^2+^/calmodulin-dependent protein kinase II α activity (Hu et al., 2015 ▶). Pretreatment with curcumin had no noteworthy effect on acute MIH, however, its chronic administration increased METH-induced sensitization of locomotor activity (Zhao et al., 2012 ▶).

Cinnamic Aldehyde (CA) or cinnamaldehyde is a natural chemical compound of cinnamon tree, with a broad range of biological properties. In our previous study, CA showed neuroprotective effects in METH-intoxicated animals and enhanced learning and cognition. The author suggested that CA exerts its protective effects through activation of the ERK pathway in the prefrontal cortex (Saeed et al., 2018 ▶). 

## Conclusion

METH is a highly addictive substance and induces many destructive effects in brain and causes psychological problems in acute or chronic toxicity. Nowadays, herbal products are very affordable, effective and available and are widely used for therapeutic purposes. Various herbal plants have been suggested for alleviating METH adverse effects and reducing tolerance and dependence to METH. Based on *in vitro* and *in vivo* findings, only a few natural products such as ginseng and levo-tetrahydropalmatine have sufficient documents and are promising candidates for treatment of METH abuse and toxicity. However, clinical trials are needed to prove these finding.
